# Quantification of cellular viability by automated microscopy and flow cytometry

**DOI:** 10.18632/oncotarget.3266

**Published:** 2015-03-09

**Authors:** Allan Sauvat, Yidan Wang, Florian Segura, Sabrina Spaggiari, Kevin Müller, Heng Zhou, Lrenzo Galluzzi, Oliver Kepp, Guido Kroemer

**Affiliations:** ^1^ Equipe 11 labellisée par la Ligue Nationale Contre le Cancer, Centre de Recherche des Cordeliers, Paris, France; ^2^ INSERM, U1138, Paris, France; ^3^ Metabolomics and Cell Biology Platforms, Gustave Roussy Cancer Campus, Villejuif, France; ^4^ Faculté de Medecine, Université Paris-Sud, Le Kremlin-Bicêtre, France; ^5^ Gustave Roussy Cancer Campus, Villejuif, France; ^6^ Faculté de Medecine, Université Paris Descartes, Sorbonne Paris Cité, Paris, France; ^7^ Pôle de Biologie, Hopitâl Européen George Pompidou, AP-HP, Paris, France

**Keywords:** apoptosis, necrosis, high-throughput screening, drug discovery

## Abstract

Cellular viability is usually determined by measuring the capacity of cells to exclude vital dyes such as 4′,6-diamidino-2-phenylindole (DAPI), or by assessing nuclear morphology with chromatinophilic plasma membrane-permeant dyes, such as Hoechst 33342. However, a fraction of cells that exclude DAPI or exhibit normal nuclear morphology have already lost mitochondrial functions and/or manifest massive activation of apoptotic caspases, and hence are irremediably committed to death. Here, we developed a protocol for the simultaneous detection of plasma membrane integrity (based on DAPI) or nuclear morphology (based on Hoechst 33342), mitochondrial functions (based on the mitochondrial transmembrane potential probe DiOC_6_(3)) and caspase activation (based on YO-PRO^®^-3, which can enter cells exclusively upon the caspase-mediated activation of pannexin 1 channels). This method, which allows for the precise quantification of dead, dying and healthy cells, can be implemented on epifluorescence microscopy or flow cytometry platforms and is compatible with a robotized, high-throughput workflow.

## INTRODUCTION

Although the molecular mechanisms that are involved in the regulation and execution of cell death have been studied extensively during the last decades, the definition of cell death itself is still a matter of debate [1, 2]. It has been proposed that the demise of proliferating cells corresponds to the loss of their clonogenic potential [3]. However, this definition cannot be extended to post-mitotic cells (*e.g*., neurons and cardiomyocytes), which definitively exited the cell cycle along with terminal differentiation [4], and is rather inaccurate, as proliferating cells can enter a temporary or irreversible cell cycle arrest (and hence fail to replicate), while maintaining biological functions (for instance the secretion of soluble mediators), for very long periods [5, 6]. Other, more accurate definitions of cell death should therefore be employed. Recently, a large committee of experts in the field agreed on pragmatically identifying the transition between a reversible perturbation of cellular homeostasis and cell death (which is irreversible, by definition) with the permanent loss of plasma membrane integrity [7]. This process can be conveniently monitored by the uptake of so-called exclusion dyes, i.e., colored or fluorescent molecules that are excluded by healthy cells owing to the integrity of their plasma membrane [2, 8]. A commonly employed exclusion dye is 4′,6-diamidino-2-phenylindole (DAPI) [8].

However, plasma membrane permeabilization is a late process in the cascade of biochemical events that bridges the delivery of a lethal stimulus to the execution of cell death [7, 9]. This implies that, at least in some instances, other processes well upstream of plasma membrane permeabilization may irreversibly commit cells to die, including the complete loss of mitochondrial functions [10–12], and the massive activation of cysteine proteases of the caspase family [13, 14]. Thus, although plasma membrane permeabilization constitutes the gold standard indicator of cell death, at least *in vitro*, there are many experimental settings in which it is important to quantify the proportion of cells that still possess an intact plasma membrane yet are doomed to die.

Mitochondrial functions are usually monitored by means of cationic lipophilic fluorochromes that accumulate within the mitochondrial matrix driven by the mitochondrial transmembrane potential (Δψ_m_), such as 3,3′-dihexyloxacarbocyanine iodide (DiOC_6_(3)) or tetramethylrhodamine ethyl ester (TMRE) [15] At odds with their functional counterparts, dysfunctional mitochondria exhibit indeed a reduced Δψ_m_, resulting in an impaired accumulation of these probes [16–18]. One economic way of monitoring caspase activation relies on YO-PRO^®^-3, a fluorochome that accumulates in the nucleus upon crossing the plasma membrane through pannexin 1 (PANX1) channels [19–21]. PANX1 channels are activated by caspase-3 before the onset of plasma membrane permeabilization, a setting in which cells do not take up vital dyes yet, but secrete small metabolites (such as ATP) and accumulate YO-PRO^®^-3 [22–24]. Of note, cells with permeabilized plasma membrane may exhibit profound nuclear alterations, including chromatin condensation and shrinkage (which are associated with apoptosis) or nuclear dilation (which is associated with necrosis). These changes can be monitored by epifluorescence microscopy using a plasma-membrane chromatinophilic dye such as Hoechst 33342 [2, 8].

Based on these premises, we decided to combine an exclusion dye (DAPI) or a dye for monitoring nuclear integrity (Hoechst 33342) (both of which emit a blue fluorescence) with DiOC_6_(3) (which emits a green fluorescence) and YO-PRO^®^-3 (which emits a red/orange fluorescence) to monitor the evolution of cells exposed to lethal stimuli. Here, we demonstrate that such a co-staining approach allows for monitoring the absolute number and percentage of truly viable cells (which exhibit an intact plasma membrane with closed PANX1 channels and high Δψ_m_). This procedure is compatible with a fully robotized workflow in which cellular samples are processed, stained and analyzed by epifluorescence microscopy or flow cytometry.

## RESULTS AND DISCUSSION

### Epifluorescence microscopy-based fine analysis of cellular viability

**Figure 1:**
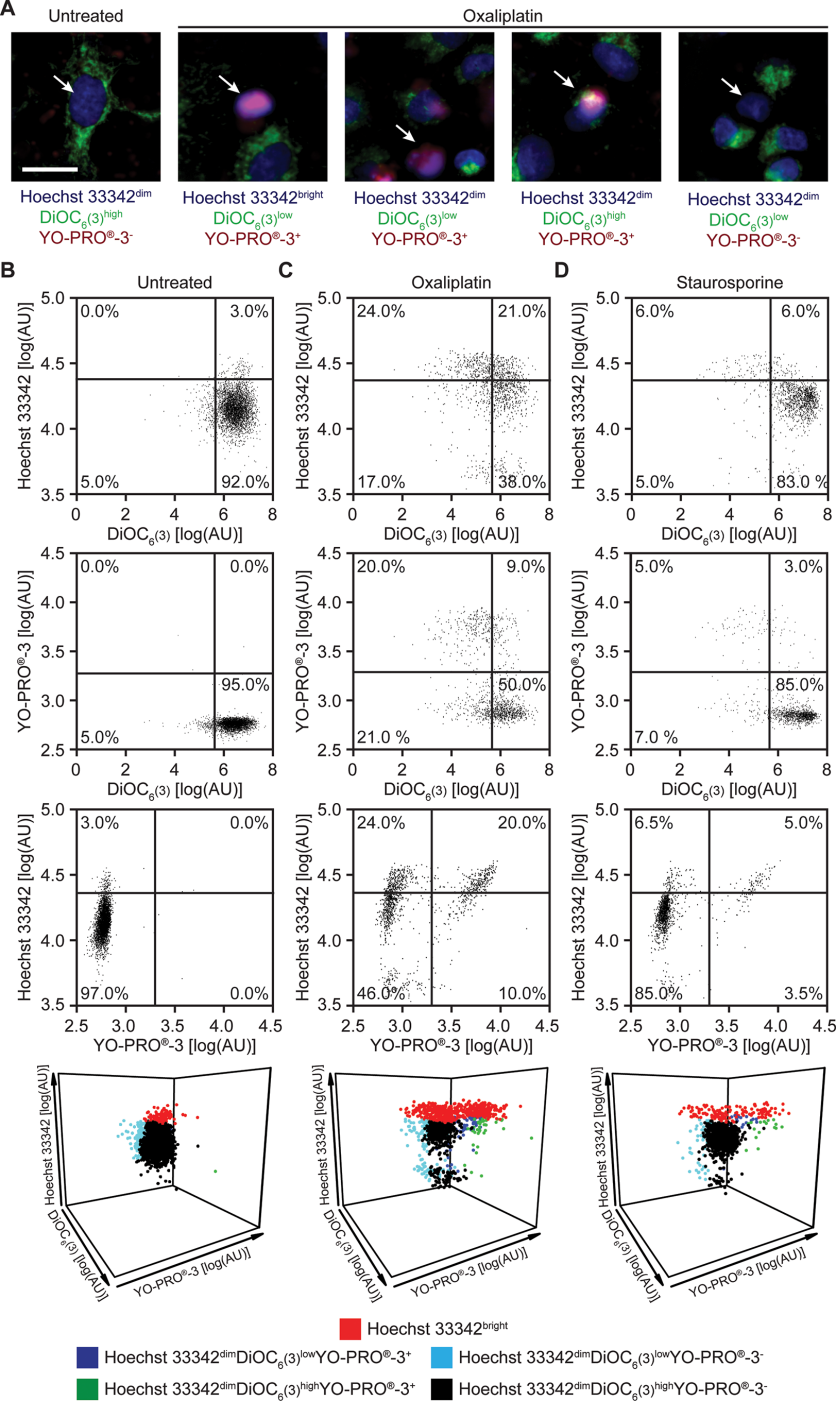
Epifluorescence microscopy-based assessment of cellular viability in response to standard inducers of apoptosis **(A–D)** Human non-small cell lung carcinoma A549 cells were maintained in control conditions or exposed to 500 μM oxaliplatin or 4 μM staurosporine for 24 hrs, then imaged by epifluorescence microscopy upon co-staining with Hoechst 33342, DiOC_6_(3) and YO-PRO^®^-3. Representative images and dot plots obtained upon automated image segmentation and analysis are reported. Scale bar = 10 μm. In panels B, C and D, numbers indicate the percentage of events within each gate. See also Supplementary Figure 1.

To develop an automatable method for the simultaneous assessment of plasma membrane integrity, mitochondrial functions and caspase activation, we maintained human non-small cell lung carcinoma (NSCLC) A549 cells in control conditions or we exposed them to a lethal dose of the multikinase inhibitor staurosporine (a well-known inducer of apoptosis) [25] or the platinum derivative oxaliplatin [26, 27] for 24 hrs. Thereafter, we simultaneously stained cells with Hoechst 33342, DiOC_6_(3) and YO-PRO^®^-3 and analyzed them by epifluorescence microscopy. Upon automated image segmentation (Supplementary Figure 1), we identified five possible cellular phenotypes: (1) cells that stained weakly for Hoechst 33342 (Hoechst 33342^dim^ cells, manifesting normal nuclear morphology) and negatively for YO-PRO^®^-3 (and hence retained normal plasma membrane impermeability) while exhibiting a high DiOC_6_(3) signal (preserving a high Δψ_m_); (2) cells that stained intensely for both Hoechst 33342 (Hoechst 33342^bright^ cells, manifesting nuclear condensation) and YO-PRO^®^-3 (manifesting a complete loss of plasma membrane barrier functions) while exhibiting a low DiOC_6_(3) signal (a sign of complete impairment of mitochondrial functions); and (3–5) Hoechst 33342^dim^ cells bearing either YO-PRO^®^-3 positivity or low DiOC_6_(3) signal, or both (three intermediate situations) (Figure 1A). In control conditions, most cells exhibited a uniform Hoechst 33342^dim^DiOC_6_(3)^high^YO-PRO^®^-3^−^ phenotype (Figure 1B and Supplementary Figure 2), a staining profile that clearly changed in response to cell death induction. Exposing A549 cells to 500 μM oxaliplatin or 4 μM staurosporine for 24 hrs, indeed, provoked an increase in the frequency of Hoechst 33342^bright^ cells as well as of Hoechst 33342^dim^DiOC_6_(3)^low^YO-PRO^®^-3^+^, Hoechst 33342^dim^DiOC_6_(3)^high^YO-PRO^®^-3^+^ or Hoechst 33342^dim^DiOC_6_(3)^low^YO-PRO^®^-3^−^ cells as it reduced the frequency and absolute number of healthy Hoechst 33342^dim^DiOC_6_(3)^high^YO-PRO^®^-3^−^ cells (Figure 1C, 1D and Supplementary Figure 2).

### Implementation of the triple straining procedure on flow cytometry

**Figure 2:**
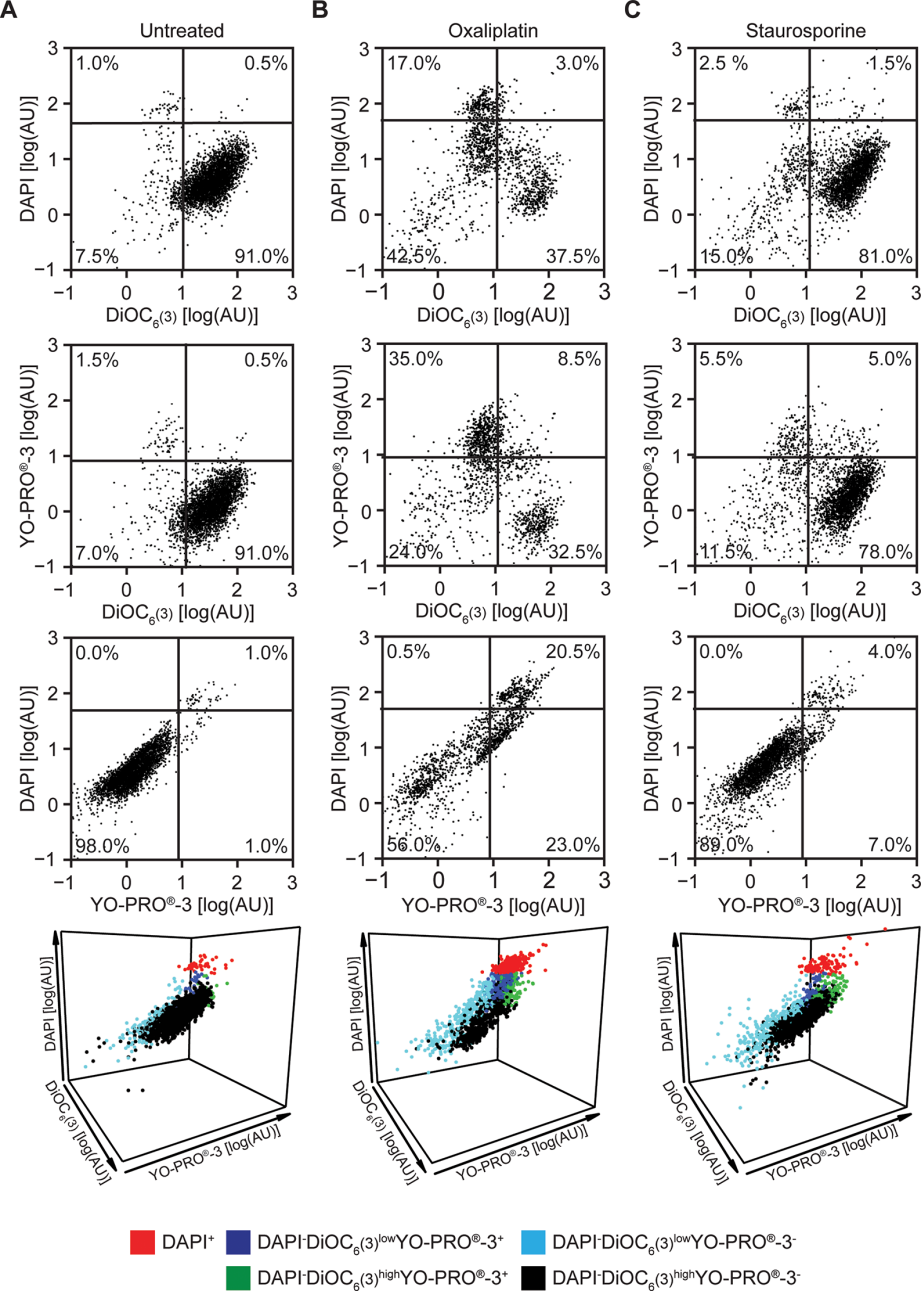
Flow cytometry-based assessment of cellular viability to standard inducers of apoptosis **(A–C)** Human non-small cell lung carcinoma A549 cells were cultured in control conditions or treated with 500 μM oxaliplatin or 4 μM staurosporine for 24 hrs, then co-stained with DAPI, DiOC_6_(3) and YO-PRO^®^-3 and analyzed by flow cytometry. Representative dot plots are reported. In panels A, B and C, numbers indicate the percentage of events within each gate. See also Supplementary Figure 2.

**Figure 3:**
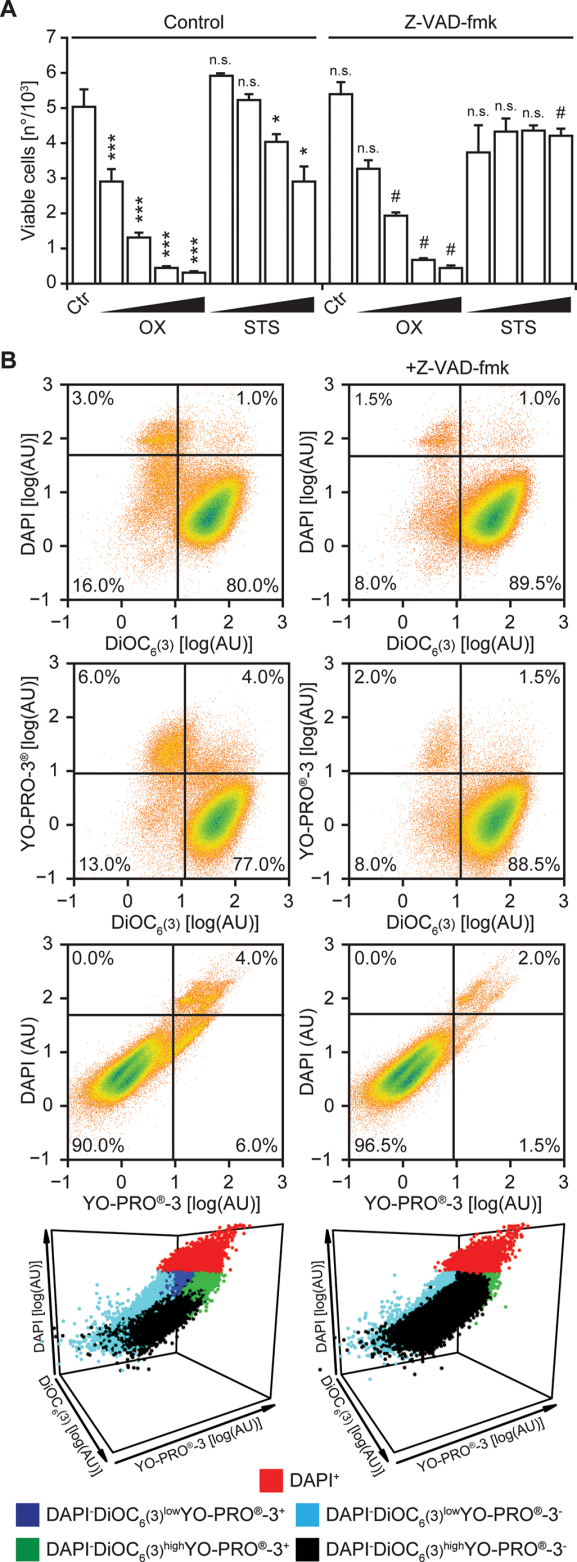
Flow cytometry-based assessment of cell death modulation **(A–B)** Human non-small cell lung carcinoma A549 cells were maintained in control conditions (Ctr) or incubated with increasing concentrations of staurosporine (STS; 0.5–1.0–2.0–4.0 μM), oxaliplatin (OX; 0.125–0.250–0.500–1 mM) and 50 μM Z-VAD-fmk, alone or in combination for 24 hrs. Thereafter, cells co-stained with DAPI, DiOC_6_(3) and YO-PRO^®^-3 and analyzed by flow cytometry. In panel **(A)** quantitative data on the absolute number of DAPI^−^DiOC_6_(3)^high^YO-PRO^®^-3^−^ (viable) cells are reported (means ± SD, *n* = 3 replicate assessments from one representative experiment; *p < 0.05, ***p < 0.001, as compared to untreated cells; ^#^*p* < 0.05, as compared to cells maintained in the same conditions in the absence of Z-VAD-fmk; n.s., non-significant, as compared to untreated cells or cells maintained in the same conditions in the absence of Z-VAD-fmk). In panel **(B)** dot plots depict the aggregate analysis of cells maintained in control conditions and exposed to 4 μM STS or 500 μM OX, alone (left panels, upon pooling data from 3 distinct samples) or in the presence of Z-VAD.fmk (right panels, upon pooling data from 3 distinct samples). Numbers indicate the percentage of events within each gate.

Next, we implemented the triple staining protocol described above on a cytofluorometric platform amenable to automation. To this aim, human NSCLC A549 cells maintained in control conditions or exposed to lethal stimuli for 24 hrs were collected and then co-stained with DAPI, DiOC_6_(3) and YO-PRO^®^-3. The vast majority of untreated A549 cells exhibited a DAPI^−^DiOC_6_(3)^high^ YO-PRO^®^-3^−^ phenotype, indicating intact plasma membranes, normal mitochondrial functions and no caspase activation (Figure 2A and Supplementary Figure 3). In contrast, exposing A549 cells to 500 μM oxaliplatin or 4 μM staurosporine for 24 hrs caused the accumulation of DAPI^+^ cells, as well as of cells exhibiting a DAPI^−^DiOC_6_(3)^low^YO-PRO^®^-3^+^, DAPI^−^DiOC_6_(3)^high^YO-PRO^®^-3^+^ or DAPI^−^DiOC_6_(3)^low^ YO-PRO^®^-3^−^ staining pattern (Figure 2B, 2C and Supplementary Figure 3). Hence, both oxaliplatin and staurosporine cause a drop in the absolute amount of DAPI^−^DiOC_6_(3)^high^ YO-PRO^®^-3^−^ (viable) A549 cells in a dose-dependent fashion (Figure 3A). The pan-caspase inhibitor N-benzyloxycarbonyl-Val-Ala-Asp(O-Me) fluoromethylketone (Z-VAD-fmk) was unable to prevent the loss of viability among cells cultured in the presence of oxaliplatin or staurosporine. However, caspase inhibition with Z-VAD-fmk substantially reduced the surge in DAPI^−^DiOC_6_(3)^low^YO-PRO^®^-3^+^ and DAPI^−^DiOC_6_(3)^high^YO-PRO^®^-3^+^ cells, without eliminating the DAPI^−^DiOC_6_(3)^low^YO-PRO^®^-3^−^ subset. This was particularly evident when all events in control, oxaliplatin or staurosporine-treated conditions were analyzed upon aggregation, as if they derived from a single sample, either in the absence (Figure 3B, left panels) or in the presence of Z-VAD-fmk (Figure 3B, right panels). These results confirm the stringent requirement for caspase activation for the opening of YO-PRO^®^-3-permeable PANX1 channels [19, 20], and validate the feasibility and sensitivity of our viability test.

### Detection of synergistic interactions by automatic viability measurements

**Figure 4:**
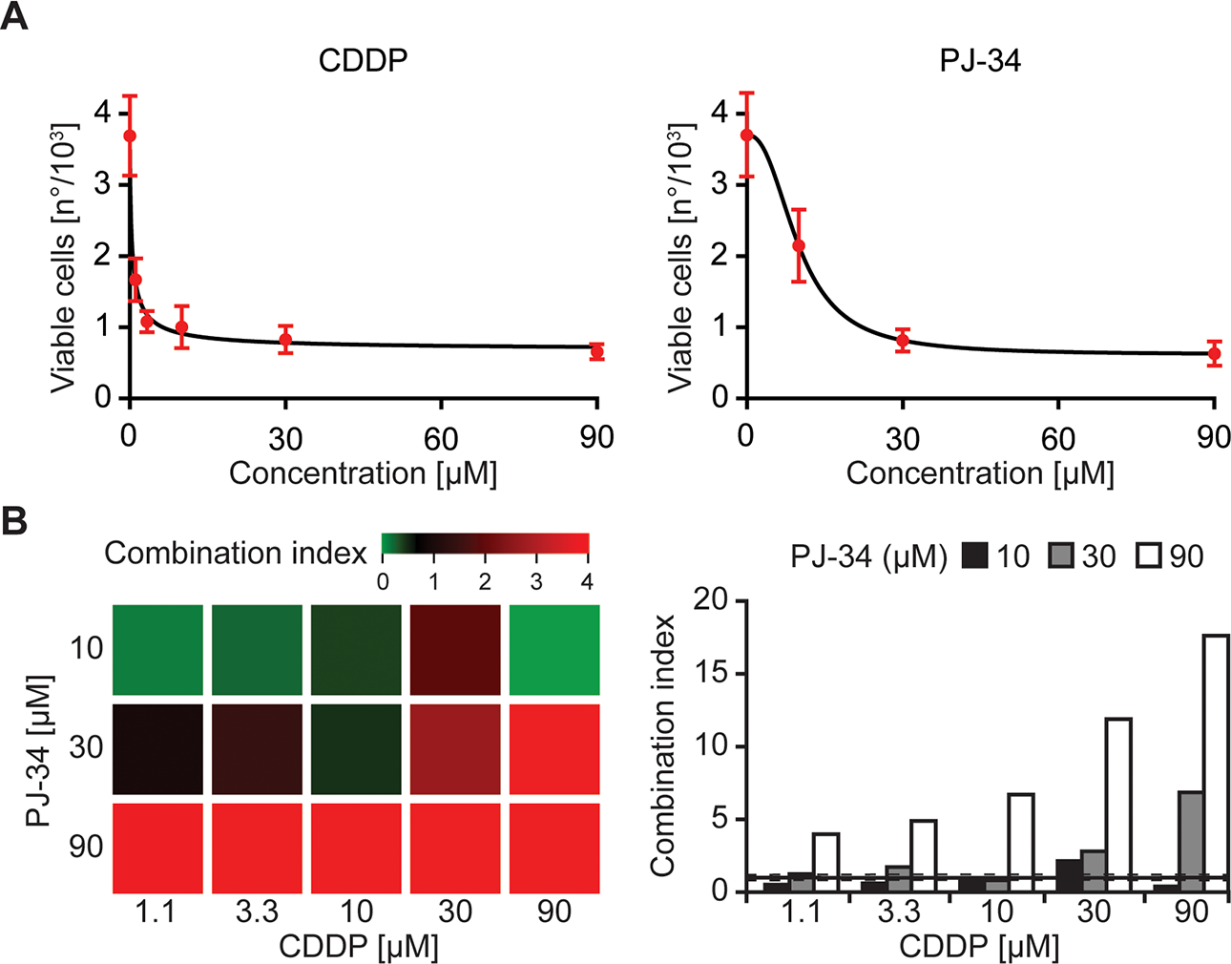
Flow cytometry-based assessment of pharmacological interactions **(A, B)** Human non-small cell lung carcinoma A549 cells were left untreated or treated with the indicated concentrations of cisplatin (CDDP) or PJ-34 for 48 hrs, then stained with DAPI, DiOC_6_(3) and YO-PRO^®^-3 and analyzed by flow cytometry. Panel A reports quantitative data on DAPI^−^DiOC_6_(3)^high^YO-PRO^®^-3^−^ (viable) cells (means ± SD, *n* = 3 replicate assessments from one representative experiment), while panel B illustrates combination indexes (CIs) calculated according the Harbron's method. Please note that CIs < 0.8 (green on the heatmap) and > 1.2 (red on the heatmap) represent synergistic and antagonistic interactions, respectively.

To further investigate the utility of our fully automated viability test, we measured its performance on the pharmacological interaction between another platinum derivative, i.e., *cis*-diamminedichloroplatinum(II) (CDDP, best known as cisplatin) and the poly(ADP-ribose) polymerase 1 (PARP1) inhibitor PJ-34 hydrochloride hydrate (hereafter referred to as PJ-34) [28, 29]. CDDP reduced the number of DAPI^−^DiOC_6_(3)^high^YO-PRO^®^-3^−^ (viable) A549 cells at much lower concentrations than did PJ-34 (Figure 4A). Moreover, at certain concentrations, CDDP and PJ-34 exhibited a synergistic interaction, meaning that their co-administration reduced the number of DAPI^−^DiOC_6_(3)^high^YO-PRO^®^-3^−^ A549 cells in a hyperadditive fashion, and hence was much more effective than the administration of either the two agents alone (Figure 4B). Importantly, these results were obtained by means of a completely automated workflow that did not require any manual intervention.

### Concluding remarks

Here, we describe a straightforward workflow that allows for the accurate and automatable quantification of living cells based on several parameters, namely, plasma membrane integrity, PANX1 channel impermeability and normal Δψ_m_. The protocol that has been developed to this aim is robust (reagents are not particularly prone to degradation or sensitive to unpredictable environmental conditions), simple (it does not involve washing steps), rapid (staining time = 30 min), can be readily scaled up (as shown for 96-well plates) and is adaptable to different readouts (as shown for epifluorescence microscopy and flow cytometry). This implies that our protocol can be implemented on virtually any robotized platform that allows for automatic pipetting (for trypsinization and staining) and incubation, and is coupled to any reader (be it an epifluorescence microscope or a flow cytometer) that can automatically handle multi-well plates.

One advantage of modern microscopic and cytofluorometric systems is that they are compatible with the quantification of the absolute number of cells per area or volume. Thus, the staining protocol described here allows for the precise discrimination of live, dead and dying cells in a variety of experimental samples, including transformed cells growing on plates or in suspension, as well as primary cells of animal or human derivation. This informs on cytostatic and/or cytotoxic effects induced by anticancer agents, promising ameliorations for future drug screening campaigns and mechanistic explorations.

## MATERIALS AND METHODS

### Chemicals and cell cultures

Except otherwise indicated, cell culture media and supplements were obtained from Gibco-Life Technologies (Carlsbad, CA, USA), chemicals from Sigma-Aldrich (St. Louis, MO, USA), and plasticware from Greiner Bio-One (Monroe, CA, USA). Human non-small cell lung carcinoma (NSCLC) A549 cells were cultured in Glutamax^®^-containing DMEM/F12 medium supplemented with 10% fetal calf serum (FCS), and 10 mM HEPES buffer. Cells were grown at 37°C in a humidified incubator generating a 5% CO_2_ atmosphere.

### Automated experimental workflow

All experimental steps were conducted on the PACRI HTS cell biology platform that integrates a Biomek FX^P^ automated liquid handling workstation (Beckman–Coulter, Fullerton, CA, USA), a multidrop combi automated dispenser (ThermoScientific, Whaltam, MA, USA), a Cytomat6000 automated cell culture incubator (ThermoScientific), 3 ImageXpress Micro XL automated microscopes (Molecular Devices, Sunnyvale, CA, USA), a CyAn ADP cytofluorometer (Beckman–Coulter), a Hypercyt loader (Intellicyt, Albuquerque, NM, USA) and a Motoman HP3JC industrial robot (Motoman, West Carrollton, OH, USA). A fully automated workflow entailing cell culture, drug treatment, sample preparation and cytofluorometric or microscopic acquisition was generated by means of the SAMI automation control software (Beckman–Coulter). Multiplex assays were prepared in parallel for microscopic and cytofluorometric acquisition.

### Cell death assays

Five × 10^3^ human NSCLC A549 cells were seeded in 96-well clear cell culture plates or black imaging plates for flow cytometry- or epifluorescence microscopy-based assays, respectively. Cells were allowed to adapt for 24 hrs and then maintained in control conditions or exposed to lethal stimuli for 24 or 48 hrs. For flow cytometry, culture supernatants were discarded and cells were detached with 30 μL TrypLE™ Express per well, then resuspended in 30 μL of medium supplemented with 40 nM DiOC_6_(3), 1 μM YO-PRO^®^-3 iodide and 2 μM DAPI (all from Molecular Probes^®^−Life Technologies, Carlsbad, CA, USA). Subsequently, cells were transferred to a 96-well V-shape plate and incubated for 30 min at 37°C before acquisition. Cytofluorometric acquisitions were performed on a CyanADP or a MACSQuant cytometer (Miltenyi Biotec, Bergisch Gladbach, Germany). For epifluorescence microscopy, supernatants were removed and cells were incubated in 30 μL of medium supplemented with 40 nM DiOC_6_(3), 1 μM YO-PRO^®^-3 iodide and 2 μM Hoechst 33342 for 30 min at 37°C before acquisition. Images were acquired on ImageXpress Micro XL automated microscopes (4 view fields per well).

### Data processing and statistical analyses

Unless otherwise specified, experiments were performed in quadruplicate parallel instances, and data were analyzed with the R software (http://www.r-project.org/). The first-line flow cytometry data analysis was performed using the flowcore package for R (http://www.bioconductor.org) upon gating on events with normal forward and side scatter parameters. Microscopy images were analyzed by means of the MetaXpress (Molecular Devices) software. In particular, images were segmented using the built-in custom module editor to identify nuclei (based on Hoechst 33342 fluorescence), and cytoplasmic regions (based on DiOC_6_(3) fluorescence). Thereafter, the average nuclear signal of Hoechst 33342 and YO-PRO^®^-3 as well as the average cytoplasmic signal of DiOC_6_(3) were measured. The pharmacological interaction between PJ-34 and CDDP was evaluated computing combination indexes (CI) following Harbron's method [30, 31]. Unless otherwise specified, data are presented as means ± SD. Statistical significance was assessed with two-tailed, unpaired Student's *t*-tests.

## SUPPLEMENTARY FIGURES


