# Supplementary material for: Quantification of cellular viability by automated microscopy and flow cytometry
Source: Oncotarget. 2015 Mar 9;6(11):9467–75. doi: 10.18632/oncotarget.3266 (PMC4496231; doi:10.18632/oncotarget.3266)
Supplement: Supplementary file 1 [file oncotarget-06-9467-s001.pdf]

SUPPLEMENTARY FIGURES

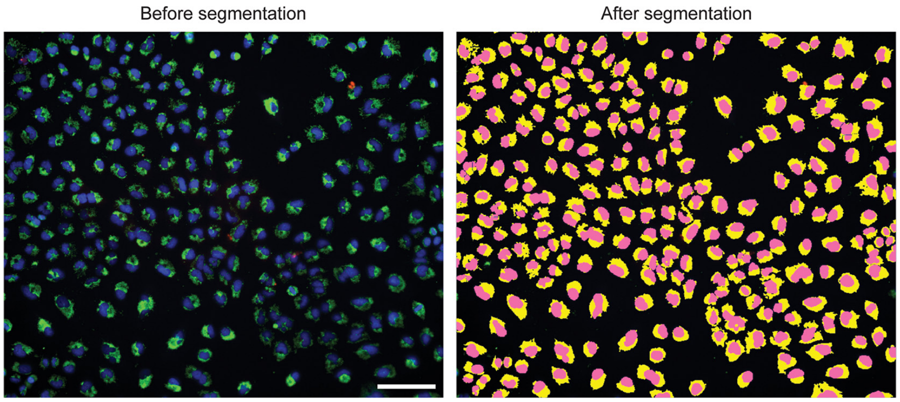

Supplementary Figure 1: Automated image segmentation. Human non-small cell lung carcinoma A549 cells maintained in control conditions for 24 hrs were fixed, then imaged by epifluorescence microscopy upon co-staining with Hoechst 33342, DiOC$_6$(3) and YO-PRO®-3. Representative images before and after segmentation are depicted. Scale bar = 50 μm.

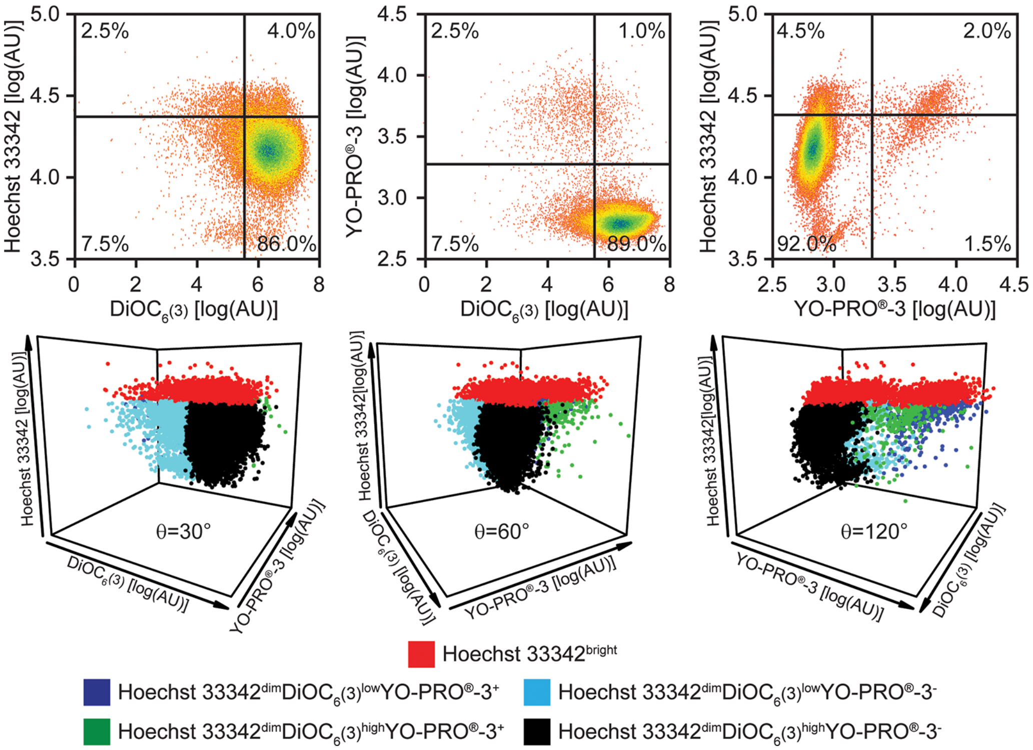

Supplementary Figure 2: Epifluorescence microscopy-based assessment of cellular viability in response to standard inducers of apoptosis. Human non-small cell lung carcinoma A549 cells were maintained in control conditions or exposed to 500 μM oxaliplatin or 4 μM staurosporine for 24 hrs, then imaged on epifluorescence microscopy upon co-staining with Hoechst 33342, DiOC$_6$(3) and YO-PRO®-3. Dot plots depicting the aggregate analysis of cells maintained in control conditions and exposed to staurosporine or oxaliplatin are reported (upon pooling data from 3 distinct samples).

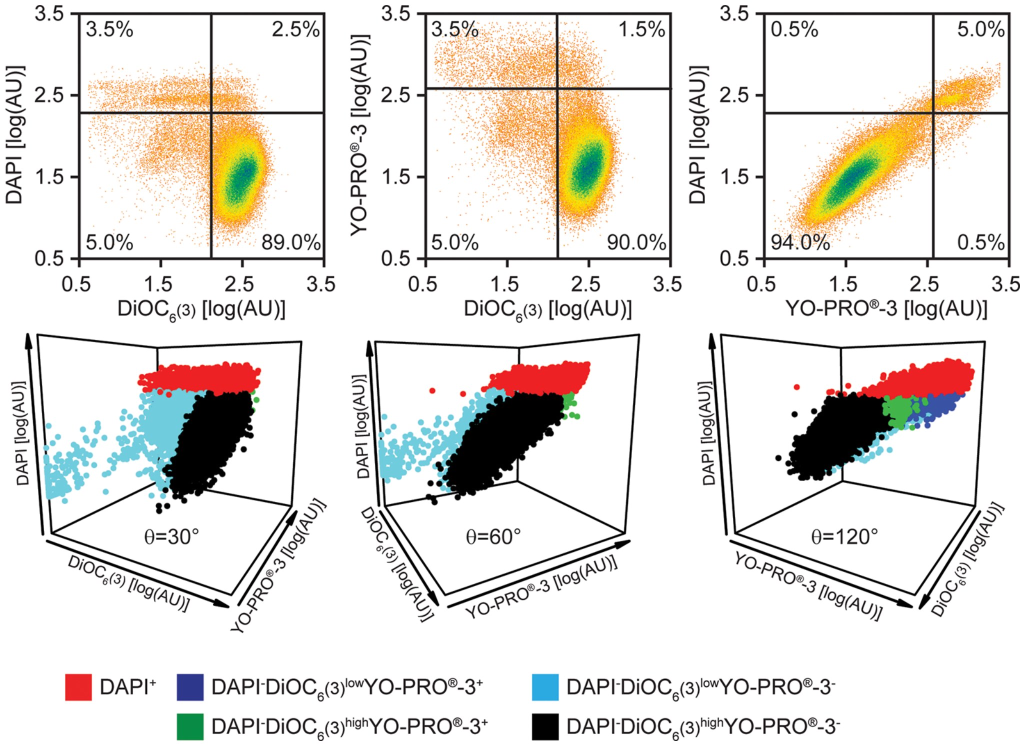

Supplementary Figure 3: Flow cytometry-based assessment of cellular viability to standard inducers of apoptosis. Human non-small cell lung carcinoma A549 cells were cultured in control conditions or treated with 500 µM oxaliplatin or 4 µM staurosporine for 24 hrs, then co-stained with DAPI, $DiOC_6(3)$ and YO-PRO®-3 and analyzed by flow cytometry. Dot plots depicting the aggregate analysis of cells maintained in control conditions and exposed to staurosporine or oxaliplatin are reported (upon pooling data from 3 distinct samples).
